# CT-Derived Fractional Flow Reserve Guides Surgical Revascularization in Anomalous Long Left Anterior Descending With Curly-Q Intramyocardial Course

**DOI:** 10.1016/j.jaccas.2025.105750

**Published:** 2025-10-29

**Authors:** Sophia Golec, Christopher Clyne, Andrei Minciunescu, Alexi Otrakji, Mohamad El-Zaru, Gregory Couper, Madhavi Kadiyala

**Affiliations:** a Tufts Medical Center, Boston, Massachusetts, USA; b Massachusetts General Hospital, Boston, Massachusetts, USA

**Keywords:** computed tomography, coronary angiography, coronary artery bypass, coronary vessel anomaly, echocardiography, fractional flow reserve, MR sequences, reduced ejection fraction, shortness of breath, three-dimensional imaging

## Abstract

**Background:**

Anomalous coronary arteries with intramyocardial courses pose diagnostic and management challenges.

**Case Summary:**

A 61-year-old woman with dyspnea, left bundle branch block, and reduced ejection fraction (25%) was found to have an anomalous long left anterior descending artery originating from the right coronary artery, coursing intramyocardially with a curly-Q configuration. Coronary computed tomography angiography revealed no obstructive disease, but computed tomography–derived fractional flow reserve showed a significant drop (0.78-0.79) distal to the curly-Q. This physiological assessment confirmed flow limitation, prompting a multidisciplinary decision to pursue coronary artery bypass grafting for revascularization. Postoperatively, the patient had symptomatic and functional improvement with improved ejection fraction and resolution of left bundle branch block.

**Discussion:**

Computed tomography–derived fractional flow reserve adds essential value in the management of anomalous coronary arteries, offering a noninvasive assessment of ischemia that can guide revascularization decisions in structurally challenging anatomy.

## Visual Summary

**Figure:**
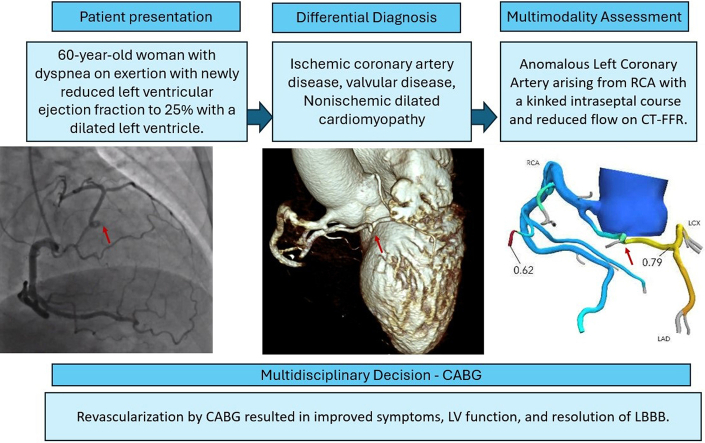

## History of Presentation

A 61-year-old woman presented with progressive exertional dyspnea which acutely worsened 2 days prior to evaluation. She described a sudden inability to take a deep breath but denied chest pain, palpitations, dizziness, or lower-extremity edema. She had long-standing exertional fatigue and limited physical activity due to shortness of breath.

**Visual Summary:**
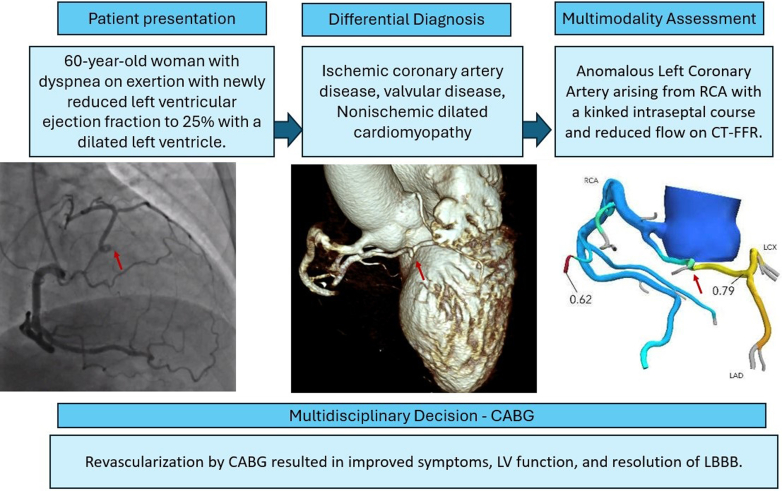
Multimodality Imaging Guided Management in Anomalous Coronary Artery Disease

## Past Medical History

Her medical history was notable for hypertension, type 2 diabetes, and obesity, with partial metabolic improvement following gastric bypass surgery 6 years earlier. She had a maternal family history of left ventricular noncompaction and a paternal history of coronary artery disease.

**Take-Home Messages**

• Anomalous coronary arteries are rare and anatomically complex, requiring individualized assessment and management strategies. Determining their hemodynamic significance is critical for guiding clinical decision-making.
• CT-derived fractional flow reserve is an emerging noninvasive tool that may aid in assessing the physiological impact of anomalous coronary anatomy and support treatment planning.

## Differential Diagnosis

Given her presentation and risk factors, primary cardiac causes were prioritized. These included heart failure with reduced ejection fraction, ischemic cardiomyopathy, and valvular heart disease. Her age, postmenopausal status, hypertension, and diabetes suggested elevated risk for coronary artery disease, which in women can present atypically with dyspnea in the absence of chest pain. Diastolic dysfunction and structural anomalies were also considered.

## Investigations

**Figure 1:**
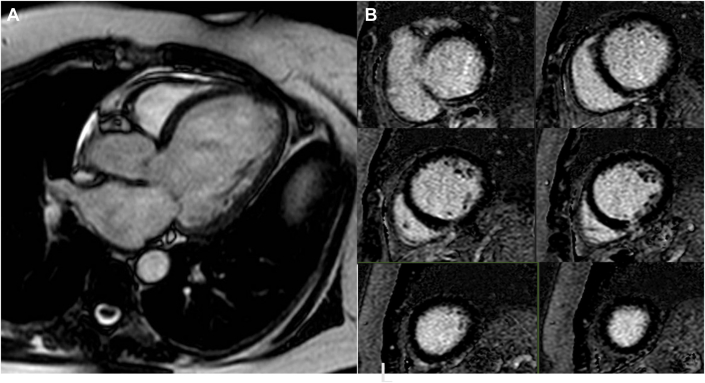
Cardiac Magnetic Resonance Imaging (A) Still frame of cardiac magnetic resonance imaging demonstrating a dilated left ventricle (indexed end diastolic volume of 126 mL/m^2^). (B) Panel of late gadolinium images demonstrates preserved viability with the absence of a scar.

The patient had an electrocardiogram which noted the presence of a left bundle branch block (LBBB). An echocardiogram demonstrated a severely dilated left ventricle with normal wall motion but an ejection fraction of 25%. Given her family history of left ventricular noncompaction, a cardiac magnetic resonance imaging exam was pursued (Figure 1). It demonstrated a severely dilated left ventricle with moderately reduced ejection fraction of 33%, diffuse global hypokinesis as well as an asynchronous interventricular septum, and normal left ventricular wall thickness. There was no significant late gadolinium enhancement. Her right ventricle was normal in size and function.

**Figure 2:**
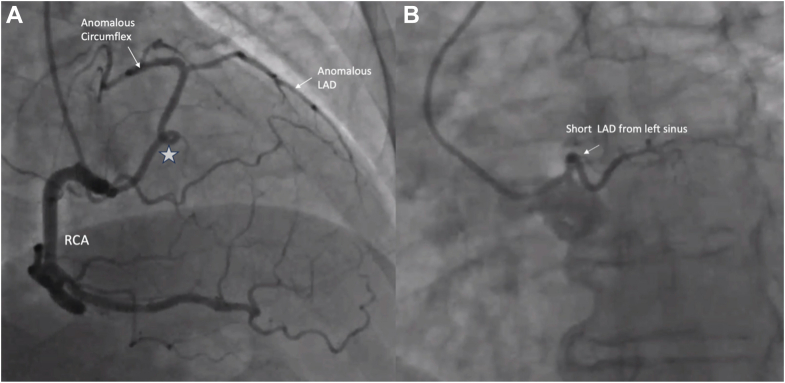
Invasive Coronary Angiography (A) The right coronary artery (RCA) was selectively engaged, revealing a dominant vessel originating from the right coronary sinus. A large anomalous proximal branch arose from the RCA and appeared to bifurcate into the left circumflex artery (LCx) and a vessel resembling the left anterior descending (LAD) artery with an identifiable diagonal branch. The anomalous vessel had a curly-Q-shaped kink in its course (star). (B) A diminutive accessory LAD with a short course along the anterior interventricular groove was noted to originate from the left coronary sinus (Video 1).

She was referred for a left- and right-heart catheterization which noted normal filling pressures and an anomalous coronary artery system (Figure 2, Video 1, Video 2, Video 3). A long left anterior descending (LAD) artery originated from the proximal right coronary artery, approximately 5 mm from the ostium.

**Figure 3:**
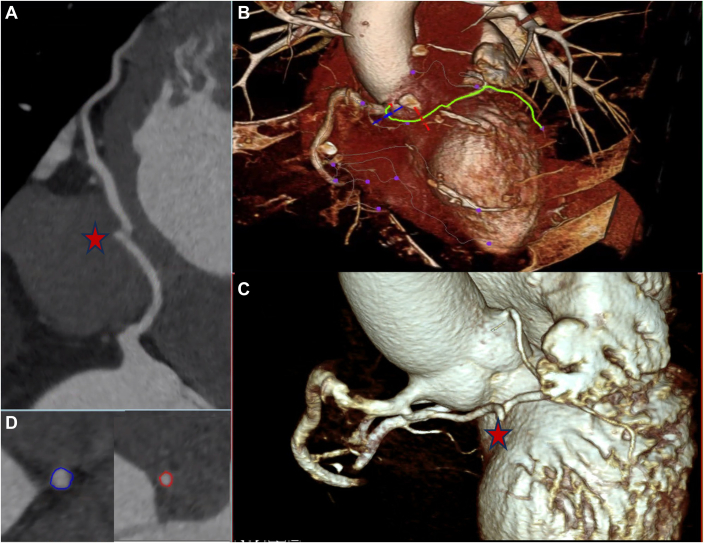
Computed Tomography Angiography of the Coronary Arteries (A) Coronary CT with curved multiplanar reformat of the anomalous coronary artery demonstrating a long intramyocardial course with a characteristic “curly-Q” configuration (red star). (B and C) Three-dimensional volume-rendered images of the coronary anatomy, shown with and without the right ventricular outflow tract for spatial reference. (D) Cross-sectional views of the arterial lumen highlighting significant narrowing (red) within the intramyocardial segment compared to the proximal segment (blue).

Further evaluation with cardiac computed tomography clarified the anomalous anatomy (Figures 3A to 3D, Video 4, Video 5, Video 6). What appeared to be the circumflex artery on catheterization was, in fact, part of an anomalous vessel with an interarterial and intraseptal course. There was an unusual curly-Q-shaped configuration within the intraseptal portion. This vessel emerged in the anterior interventricular groove and branched off into the true LAD and the left circumflex vessel. An accessory short LAD was noted to originate from the left coronary cusp, terminating early in the proximal anterior interventricular groove.

**Figure 4:**
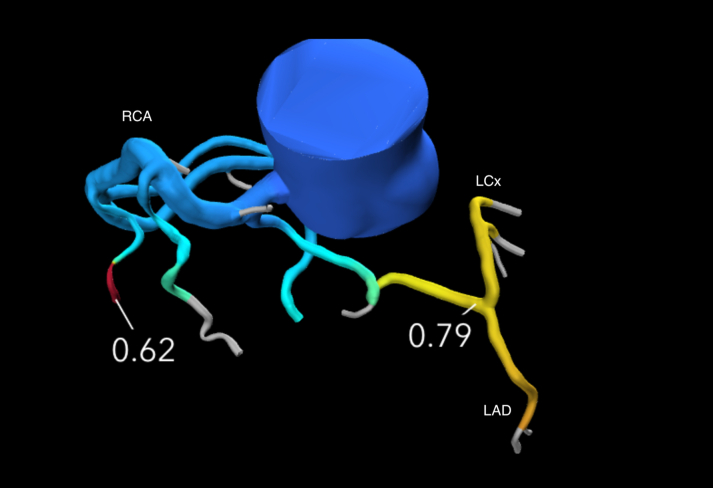
CT-FFR Analysis Demonstrated a Drop in Fractional Flow Reserve Distal to the “Curly-Q” Kink in the Anomalous Left Anterior Descending Artery, Indicating Physiologic Significance of the Lesion

CT-derived fractional flow reserve (CT-FFR) demonstrated a significant drop to 0.79 distal to the curly-Q configuration within the septal crest, suggestive of ischemia (Figure 4). No obstructive epicardial plaque was identified on either coronary computed tomography angiography or invasive angiography.

## Management

The patient continued to have progressive symptoms along with left ventricular dilation and reduced ejection fraction, which were attributed to compromised coronary flow from an anomalous LAD artery. Although medical therapy was considered, it was unlikely to provide durable benefit given the severe structural anomaly. The patient's preserved functional status and otherwise favorable surgical profile supported a decision to proceed with revascularization. Unroofing was initially contemplated but ultimately deemed high risk. Coronary CT demonstrated that the long LAD had a spiral intramyocardial and intraseptal course, alternating in proximity to both the right and left ventricular endocardial surfaces. Surgical unroofing in this context would be technically challenging and posed a risk of multiple septal defects. Following multidisciplinary discussion, coronary artery bypass grafting (CABG) was preferred to address the ischemic territory. Intraoperatively, the LAD was difficult to visualize and required extensive dissection through epicardial fat. A single-vessel bypass was performed, anastomosing the left internal mammary artery graft to the LAD artery.

## Outcome and Follow-Up

The patient had significant improvement of her symptoms following completion of cardiac rehabilitation post cardiac surgery. She noted an improvement in her shortness of breath and exercise capacity. Her repeat cardiac CT demonstrated almost complete normalization of her left ventricular size (Video 7). She was maintained on appropriate guideline-directed medical therapy. Although cardiac resynchronization therapy was initially considered, it was deferred as her electrocardiogram showed QRS narrowing and resolution of the LBBB. Her left ventricular ejection fraction improved from 35% to 40%.

## Discussion

Anomalous origin of the coronary arteries is a rare congenital defect that presents a clinical challenge when hemodynamically significant. Identifying high-risk anatomical features is essential, as select patients may benefit from surgical intervention. Yet, the pathophysiologic mechanisms in this setting remain incompletely defined. Proposed contributors include a slit-like ostium, an acute takeoff angle, and vessel kinking or narrowing.^1^ These features may create fixed flow limitation analogous to luminal narrowing seen in atherosclerotic coronary artery disease.

When originating from the right sinus, anomalous coronary arteries may follow an interarterial course between the aorta and pulmonary artery, a high-risk anatomical feature due to the potential for dynamic compression. Surgical repair is generally indicated for an interarterial course of the left coronary artery, even in asymptomatic individuals, given the associated risk of sudden cardiac death.^2^ In contrast, an interarterial course of the right coronary artery carries a lower risk but may warrant intervention if the patient is symptomatic or has evidence of inducible ischemia.^2^ In our patient, the presence of an interarterial course supported the need for surgical correction.

Myocardial bridging, often considered benign, can lead to clinically significant ischemia, especially when the bridged segment is both deep (>2 mm) and long (>2.5 cm).^3^ Ischemia in this setting results from dynamic systolic compression and delayed early diastolic relaxation of the tunneled segment, which limits coronary perfusion during periods of tachycardia or increased inotropy.

There are currently no universally accepted guidelines for managing patients with anomalous coronary arteries. However, certain anatomical features such as a long intramyocardial course have been associated with an increased risk of ischemic events and sudden cardiac death.^4^ For patients with long intramyocardial segments, unroofing is typically preferred due to its association with symptomatic improvement.^1^ While unroofing alleviates dynamic compression during stress, it does not address static lesions such as a slit-like orifice or acute angulation of the coronary takeoff. CABG, which bypasses both static and dynamic obstructions, may be considered in select cases. However, native competitive flow can lead to graft failure, and in some cases, ligation of the anomalous artery is performed to improve graft patency.^1^

This case illustrates the real-world challenges of managing patients with anomalous origin of a coronary artery. The patient's left ventricular dilation and reduced systolic function were consistent with hemodynamic compromise related to the complex course of the anomalous left coronary artery. While coronary artery unroofing was considered, coronary CT provided important anatomic details regarding the intraseptal course of the anomalous LAD and the associated risk of multiple septal defects with an unroofing procedure. CT-FFR provided additional physiologic data supporting the functional significance of the complex and tortuous anomalous course. The abnormal CT-FFR indicated the presence of resting ischemia, which supports the notion that the lesion was hemodynamically significant even in the absence of stress. The presence of resting ischemia may also favor long-term graft patency, as grafts placed to truly ischemic territories tend to remain functional longer. Taken together, these findings guided the multidisciplinary decision to proceed with CABG rather than unroofing.

CT-FFR is a noninvasive technology that leverages computational fluid dynamics-based artificial intelligence modeling to extract physiologic information from standard coronary CT angiography images. It has been extensively validated for assessing the physiologic significance of coronary stenosis in patients with atherosclerotic coronary artery disease. Recent studies have shown a moderate correlation between contemporary CT-FFR and invasively measured FFR, supporting its clinical utility in guiding revascularization decisions.^5^ However, its use in patients with anomalous coronary artery origins remains investigational. Early case reports, particularly in pediatric populations, suggest that abnormal CT-FFR values may correlate with high-risk anatomical features.^6^ In one such case, CT-FFR supported a conservative approach in the absence of physiological flow limitation.^7^ Its noninvasive nature offers a particular advantage in evaluating complex or intramyocardial courses, where invasive FFR may be technically challenging or high risk. Further research is needed to validate its role in the assessment and management of anomalous coronary anatomy.

## Conclusions

We present a case of a patient with an anomalous left coronary artery originating from the right coronary artery system, with a deep and tortuous intraseptal course, who presented with acute-on-chronic heart failure and reduced ejection fraction. Cardiac CT-FFR was instrumental in the complex clinical decision-making process, revealing reduced coronary flow distal to a kink in the anomalous vessel. The patient underwent CABG, resulting in symptomatic improvement, reverse remodeling with reduction in left ventricular dilatation, and resolution of LBBB.

## Funding Support and Author Disclosures

Dr Kadiyala is a consultant for HeartFlow, Inc, a company that provides physiology-based assessments using CT-FFR and has disclosed this financial relationship. All other authors have reported that they have no relationships relevant disclosures to report.

## Appendix

Video 1 Right Anterior Oblique Cranial View of the Right Coronary Artery With Anomalous Left Coronary System

Video 2 Left Anterior Oblique Cranial View of the Right Coronary Artery With Anomalous Left Coronary System

Video 3 Anteroposterior View of the Diminutive Native Left Accessory Left Anterior Descending

Video 4 Three-Dimensional Volume-Rendered Computed Tomography Images Shown With and Without the Right Ventricular Outflow Tract, Illustrating the Spatial Relationship of the Anomalous Left Coronary Artery With Other Cardiac Structures

Video 5 Axial Computed Tomography Images Demonstrate a Brief Interarterial Course as Well as an Intraseptal Course of the Anomalous Left Anterior Descending Artery

Video 6 Sagittal CT Images of the Anomalous Left Coronary Arterial System

Video 7 Cine Multiplanar Reformatted Images of Pre-Coronary Artery Bypass Grafting and Post-CABG Computed Tomography in 2 Chamber View Demonstrating Improvement in Left Ventricular Ejection Fraction.
